# Ceragenins exhibit bactericidal properties that are independent of the ionic strength in the environment mimicking cystic fibrosis sputum

**DOI:** 10.3389/fmicb.2023.1290952

**Published:** 2023-11-17

**Authors:** Karol Skłodowski, Łukasz Suprewicz, Sylwia Joanna Chmielewska-Deptuła, Szczepan Kaliniak, Sławomir Okła, Magdalena Zakrzewska, Łukasz Minarowski, Robert Mróz, Tamara Daniluk, Paul B. Savage, Krzysztof Fiedoruk, Robert Bucki

**Affiliations:** ^1^Department of Medical Microbiology and Nanobiomedical Engineering, Medical University of Białystok, Białystok, Poland; ^2^Holy Cross Cancer Center, Kielce, Poland; ^3^Institute of Health Science, Collegium Medicum, Jan Kochanowski University of Kielce, Kielce, Poland; ^4^2nd Department of Lung Diseases and Tuberculosis, Medical University of Bialystok, Bialystok, Poland; ^5^Department of Chemistry and Biochemistry, Brigham Young University, Provo, UT, United States

**Keywords:** ceragenins, antimicrobial peptides, cystic fibrosis, sodium chloride, sputum scope statement

## Abstract

The purpose of the work was to investigate the impact of sodium chloride (NaCl) on the antimicrobial efficacy of ceragenins (CSAs) and antimicrobial peptides (AMPs) against bacterial and fungal pathogens associated with cystic fibrosis (*CF*) lung infections. *CF*-associated bacterial (*Pseudomonas aeruginosa*, *Ochrobactrum* spp., and *Staphylococcus aureus*), and fungal pathogens (*Candida albicans*, and *Candida tropicalis*) were used as target organisms for ceragenins (CSA-13 and CSA-131) and AMPs (LL-37 and omiganan). Susceptibility to the tested compounds was assessed using minimal inhibitory concentrations (MICs) and bactericidal concentrations (MBCs), as well as by colony counting assays in *CF* sputum samples supplemented with various concentrations of NaCl. Our results demonstrated that ceragenins exhibit potent antimicrobial activity in *CF* sputum regardless of the NaCl concentration when compared to LL-37 and omiganan. Given the broad-spectrum antimicrobial activity of ceragenins in the microenvironments mimicking the airways of *CF* patients, ceragenins might be promising agents in managing *CF* disease.

## 1 Introduction

Cystic fibrosis (*CF*) is an inherited disorder characterized by the malfunction of the *CF* transmembrane conductance regulator (CFTR) protein, which plays an essential role in maintaining the balance of salt and water in airway epithelial cells (Saint-Criq and Gray, 2017). Due to the loss or impairment of this function in *CF*, the passage of chloride and sodium ions across the cell membrane is impaired, producing thick, sticky mucus (Boucher, 2004). Compared to healthy individuals, *CF* sputum contains about 5% less water, as well as a higher concentration of several mucins, anionic polyelectrolytes such as DNA, actin, and increased concentrations of proteases, resulting in the presence of very viscous mucus in the airways (Bhat et al., 1996; Lapierre et al., 2017; McKelvey et al., 2020; Akkerman-Nijland et al., 2021). The accumulation of mucus and impaired mucosal clearance create a favorable environment for bacterial and fungal colonization, causing chronic and recurrent infections that significantly affect the quality of life and survival of *CF* patients (Bhagirath et al., 2016; Turcios, 2020).

Furthermore, *Pseudomonas aeruginosa* and other *CF* pathogens produce highly antibiotic-tolerant biofilms that significantly impair the treatment of *CF* patients (Pragman et al., 2016; Simonin et al., 2019). Up to 95% of *CF* individuals not treated with CFTR potentiators and modulators, struggle with respiratory failure due to chronic bacterial infections accompanied by airway inflammation (Lyczak et al., 2002; Schaupp et al., 2023).

Cationic antimicrobial peptides (AMPs) are components of the innate immune response and serve as the first line of defense against pathogens. Broad-spectrum antimicrobial activity and immunomodulatory properties make AMPs promising candidates for antimicrobial agents (Rossi et al., 2008; Mansour et al., 2014; Lei et al., 2019). For instance, cathelicidin LL-37 is an endogenous AMP released from hCAP-18 protein that is synthesized by a variety of cell types, including mucosal epithelial cells, and immune cells, (Majewski et al., 2018) while omiganan is a new synthetic cationic peptide, consisting of 12 amino acids (Javia et al., 2022).

However, AMPs have certain limitations, such as susceptibility to protease or a high salt concentration, and a high propensity to interact with various extracellular matrix components (ECM), compromising their antimicrobial activity and widespread use as antimicrobial agents (Ng et al., 2017; McKelvey et al., 2020). For example, the high ionic strength and decreased pH of the surface liquid in *CF* lungs, via disruption of hydrogen bonding patterns, are potent AMPs inhibitors (Anderson and Yu, 2005; Kandasamy and Larson, 2006). Likewise, their direct interactions with highly abundant *CF* sputum and negatively charged biopolymers, including DNA, F-actin (Tang et al., 2005), and mucins (Felgentreff et al., 2006), lead to the formation of large and elongated aggregates.

Synthetic ceragenins (CSAs) are non-peptide analogs of natural AMPs that retain their antimicrobial properties while addressing some drawbacks, leading to an extended half-life in body fluids and tissues (Leszczyńska et al., 2011; Pollard et al., 2012; Sinclair et al., 2012; Durnaś et al., 2017). In addition, ceragenins show reduced potential for resistance induction, likely due to multiple mechanisms of action, including permeabilization of the microbial cell membranes and induction of reactive oxygen species. Therefore, ceragenins possess a broad spectrum of microorganisms, including multidrug-resistant (MDR) strains growing in planktonic and biofilm forms (Chmielewska et al., 2020; Paprocka et al., 2022; Tokajuk et al., 2022). Ceragenins have also demonstrated immunomodulatory and antiviral effects, making them promising candidates for versatile therapeutic applications (Howell et al., 2009; Bucki et al., 2015; Suprewicz et al., 2023).

The introduction of CFTR modulators has undoubtedly been a groundbreaking achievement in the management of *CF*, alleviating the underlying defect and substantially improving clinical outcomes, including reductions in infectious complications (Haq et al., 2022). However, continued research into new antimicrobial strategies is essential, as not all *CF* patients respond equally to CFTR modulators, and some continue to face persistent and difficult infections (McGarry et al., 2022). For instance, individuals with CFTR mutations, such as Phe508del, may not experience significant improvements in lung function or other symptoms, and infectious complications in the airways continue to pose a substantial challenge (Habib et al., 2019). Preventing the latter requires the prolonged use of antibiotics, which has led to the emergence of drug-resistant strains, underscoring the urgency for novel antimicrobial strategies. In addition, CFTR modulators may cause side effects, such as gastrointestinal and respiratory issues or headaches, impairing patients’ quality of life (Dagenais et al., 2020). Moreover, the cost of CFTR modulators may be a barrier to access for some patients or healthcare systems, thereby limiting this treatment to the world’s wealthiest nations (Zampoli et al., 2023). Finally, although short-term studies indicate that CFTR modulators improve lung function and quality of life, their long-term effects are still under investigation (Taylor-Cousar et al., 2023). Therefore, research and development of innovative antimicrobial strategies to ensure the health of individuals with *CF* is far from complete. This study evaluated the antimicrobial efficacy of ceragenins and AMPs against *CF*-associated pathogens in *CF* sputum samples alone and supplemented with NaCl excess.

## 2 Materials and methods

### 2.1 Bacterial strains

The following reference bacterial and fungal strains were tested in the study: *Staphylococcus aureus* Xen29, (Caliper Life Science Inc., Hopkinton, MA, USA), *Pseudomonas aeruginosa* ATCC 27853 (non-mucoid strain) (ATCC, Manassas, VA, USA), and *Candida albicans* ATCC 10231 (ATCC, Manassas, VA, USA). In addition, in experiments with artificially contaminated sputum, *P. aeruginosa* PAO1 DSM 19880 strain (mucoid strain) (DSMZ, DSMZ, Germany Germany) with inserted pMF230 plasmid encoding a beta-lactamase gene ensuring resistance to carbenicillin (Nivens et al., 2001) was used to differentiate it from *P. aeruginosa* (susceptible to carbenicillin) present in sputum sample collected from *CF* patient (see below). The pMF230 plasmid (Addgene, Watertown, MA, USA) was electroporated into *P. aeruginosa* PAO1 DSM 19880 using MicroPulser Electroporator (BioRad, Hercules, CA, USA) according to the procedure described by Choi et al. (2006), and transformants were further selected and maintained on LB agar plates with carbenicillin (400 μg/mL).

In addition, single clinical isolates of *P. aeruginosa* 4B (mucoid strain), *Ochrobactrum* spp. 10B, *C. albicans* 12B, and *C. tropicalis* 178 collected at the Department of Medical Microbiology and Nanobiomedical Engineering in Bialystok, Poland, were included in the study. *S. aureus*, *P. aeruginosa*, *Ochrobactrum* spp., and *Candida* strains were cultured and maintained on the recommended selective media purchased from Biomaxima (Lublin, Poland), i.e., Chapman, Cetrimide, and Sabouraud dextrose agar with chloramphenicol, respectively.

### 2.2 Compounds and experimental settings

Ceragenins were synthesized as described previously (Ding et al., 2002), whereas LL-37 and omiganan were commercially purchased from Lipopharm company (Gdańsk, Poland). The ceragenins and AMPs were dissolved in deionized water to ensure the absence of NaCl in the solution. The impact of NaCl concentrations on the antimicrobial activity of ceragenins and AMPs against the bacteria and fungi was analyzed using Mueller–Hinton (MH) broth, RPMI medium supplemented with D-(+)-glucose, and MOPS and deionized water. To that end, NaCl (Chempur, Piekary Śląskie, Poland) was suspended in these media to the final concentrations of 20 mM, 60 mM, 100 mM, 150 mM, and 300 mM. In addition, sputum samples from four cystic fibrosis patients, one positive for *P. aeruginosa*, one positive for *S. aureus*, the third positive for *P. aeruginosa, Aspergillus* and methicillin-resistant *Staphylococcus aureus* (MRSA) and the fourth positive for *C. albicans*, were included in the respective experiments in conditions mimicking *CF* lungs. For this purpose, sputum samples were diluted with deionized water to obtain 10 and 20% sputum solutions and used alone or supplemented with 150 mM or 300 mM NaCl.

### 2.3 Susceptibility testing

Microbial susceptibility testing was carried out using the serial microdilution method by current EUCAST (European Committee on Antimicrobial Susceptibility Testing) recommendations on 96-well microtiter plates with final volumes of 200 μL. For bacteria, minimum inhibitory concentrations (MICs) were determined in Mueller-Hinton broth (Sigma-Aldrich, Burlington, MA, USA), and minimum bactericidal concentrations (MBCs) were determined by placing 10 μL dilutions with no visible growth in the MIC test on the appropriate selective agar medium. For fungi, MIC was determined in 2xRPMI medium (Sigma-Aldrich, Burlington, MA, USA) supplemented with D-(+)-glucose (Sigma-Aldrich, Burlington, MA, USA) and MOPS (Sigma-Aldrich, Burlington, MA, USA) diluted twice with deionized water, while minimum fungicidal concentration (MFC) was determined by plating each sample (10 μL) on Sabouraud dextrose agar with the chloramphenicol. The final microbial concentration in the well was approximately 5 × 10^5^ CFU (colony-forming units)/mL. MIC, MBC, and MFC values were determined after 24 h incubation.

### 2.4 Killing assay

A killing assay (colony counting assay) was performed to determine the bactericidal and fungicidal activity of LL-37, omiganan, CSA-13, and CSA-131 against selected clinical strains of *P. aeruginosa* and *C. tropicalis*. Briefly, individual colonies of bacteria and fungi were resuspended at ~10^8^ CFU/mL and diluted to 10^5^ CFU/mL in sterile deionized water. Tests were performed using the AMPs and ceragenins in the 1–100 μg/mL concentration range. After 60 min of incubation at 37°C, the plates were transferred to ice, and samples were serially diluted from 10 to 1,000 times. Then 10 μL aliquots of each dilution were placed on Luria-Bertani (LB) low-salt agar containing 400 μg/mL carbenicillin (Sigma-Aldrich, Burlington, MA, USA) or on Sabouraud dextrose agar with chloramphenicol and incubated overnight at 37°C to determine the number of visible colonies. The addition of carbenicillin to LB agar selected *P. aeruginosa* PAO1 (resistant to carbenicillin) and inhibited the growth of carbenicillin-susceptible to *P. aeruginosa* present in the sputum. The colony-forming units (CFU/mL) of each sample were determined by the dilution factor.

Furthermore, the above procedure was repeated using sterile deionized water with 150 mM and 300 mM NaCl as well as 10 and 20% sputum collected from *CF* patients alone and supplemented with 150 mM and 300 mM NaCl. Due to higher bacterial survival in the presence of 20% compared to 10% sputum, the 20% sputum was selected for further experiments. After incubating the sputum samples 2 μL of Sputasol (Oxoid, Basingstoke, Hampshire, UK) was added to liquefy them, followed by the pathogens counting on the selective agars.

### 2.5 Optical microscopy and DNA measurement

DNA concentration was quantified by absorbance at 260 nm using a NanoDrop One spectrophotometer (Thermo Fisher Scientific, Waltham, MA, USA). In our study of sputum samples from cystic fibrosis patients, YOYO-1 staining (Invitrogen, Carlsbad, CA) was used to visualize DNA at a final concentration of 1 μM. Additionally, F-actin was visualized using Rhodamine Phalloidin (Thermo Fisher Scientific, Waltham, MA, USA) at a final concentration of 0.15 μM. Fluorescence images were recorded using a Leica DMi8 microscope (Wetzlar, Germany).

### 2.6 Statistical analysis

All statistical analyses were conducted using Graph Pad Prism, version 8 (GraphPad Software, Inc., San Diego, CA). The data collected were reported as the mean ± standard deviation (SD) of three experiments. The significance of differences was determined using the two-tailed Student’s test and a *p*-value ≤0.05 was considered to be statistically significant. All results were compared to the control, which were samples treated only with CSAs: CSA-13 and CSA-131, and AMPs: LL-37 and omiganan for each inducible concentration.

## 3 Results

### 3.1 The antimicrobial activity of ceragenins is not affected by high NaCl concentration

The antimicrobial activity of CSA-13 and CSA-131 against all tested bacterial and fungal strains was not affected by increasing NaCl concentration (Table 1 and Table 2), since variation in the MIC and MBC values was limited to only one dilution. On the contrary, NaCl at a concentration of 300 mM elevated 8-fold MIC and ≥ 8-fold MBC values for LL-37 and *P. aeruginosa* PAO1 as well as 8-fold MIC and ≥ 16-fold MBC values for *Ochrobactrum* spp. 10B (Table 1). A similar increase in MIC/MBC values was also noted for omiganan and *C. tropicalis* 178 (Table 2), where the MIC value increased from 16 μg/mL in the absence of salt concentration to 64 μg/mL at 300 mM NaCl, and the MFC increased from 32 μg/mL to 128 μg/mL, respectively.

**Table 1:** Minimum inhibitory concentration (MIC) and minimum bactericidal concentration (MBC) values of CSA-13, CSA-131, and LL-37 against *Staphylococcus aureus* Xen29, and *Pseudomonas aeruginosa* ATCC 27853 and DSM 19880, and two clinical isolates – *P. aeruginosa* 4B and *Ochrobactrum* spp. 10B in the presence of NaCl concentration (0–300 mM).

Compound	NaCl [mM]	MIC/MBC [μg/mL]				
		*Staphylococcus aureus* Xen29	*Pseudomonas aeruginosa* ATCC 27853	*Pseudomonas aeruginosa* PAO1 DSM 19880	*Pseudomonas aeruginosa* 4B	*Ochrobactrum* spp. 10B
CSA-13	0	0.5/0.5	2/4	2/2	1/2	1/2
	20	0.5/0.5	2/4	2/2	1/2	1/2
	60	0.5/0.5	2/4	2/4	1/2	1/2
	100	0.5/0.5	2/4	2/4	1/2	1/2
	150	0.5/0.5	2/4	2/4	1/2	1/2
	300	0.5/0.5	2/4	2/4	1/4	1/4
CSA-131	0	0.5/1	2/4	1/1	2/2	0.5/1
	20	0.5/1	2/4	1/1	2/4	0.5/1
	60	0.5/1	2/4	1/1	2/2	0.5/1
	100	0.5/0.5	2/4	1/2	2/2	0.5/1
	150	0.5/0.5	2/4	1/1	2/4	0.5/0.5
	300	0.5/0.5	2/4	1/1	2/4	0.5/1
LL-37	0	>256/>256	128/128	16/32	128/128	8/16
	20	-*	-	16/64	-	8/32
	60	-	-	16/64	-	8/32
	100	-	-	32/64	-	16/32
	150	-	-	64/128	-	16/64
	300	-	-	128/>256	-	64/>256

^*^ - not determined.

**Table 2:** Minimum inhibitory concentration (MIC) and minimum fungicidal concentration (MFC) values of CSA-13, CSA-131, and omiganan against *Candida albicans* ATCC 10231, and two clinical isolates – *C. albicans* 12B and *C. tropicalis* 178 in the presence of NaCl concentration (0–300 mM).

Compound	NaCl [mM]	MIC/MFC [μg/mL]		
		*Candida albicans* ATCC 10231	*Candida albicans* 12B	*Candida tropicalis* 178
CSA-13	0	4/4	4/4	0.25/0.5
	20	4/4	4/4	0.25/0.25
	60	4/4	4/4	0.5/0.5
	100	4/4	4/4	0.5/0.5
	150	4/8	4/4	0.5/0.5
	300	4/8	4/4	0.5/0.5
CSA-131	0	1/1	2/2	0.5/0.5
	20	1/1	2/2	0.5/0.5
	60	1/2	2/2	0.5/0.5
	100	1/1	2/2	0.5/0.5
	150	1/2	2/2	0.5/0.5
	300	1/1	2/2	0.5/0.5
Omiganan	0	>256/>256	>256/>256	16/32
	20	-*	-	16/32
	60	-	-	16/32
	100	-	-	16/32
	150	-	-	32/64
	300	-	-	64/128

^*^ - not determined.

### 3.2 Ceragenins retain antimicrobial activity in *CF* sputum and NaCl excess

A killing assay was performed to evaluate the potential inhibitory effect on ceragenins and AMPs of *CF* sputum (diluted to 10 and 20%) alone and supplemented with 150 mM and 300 mM NaCl.

**Figure 1:**
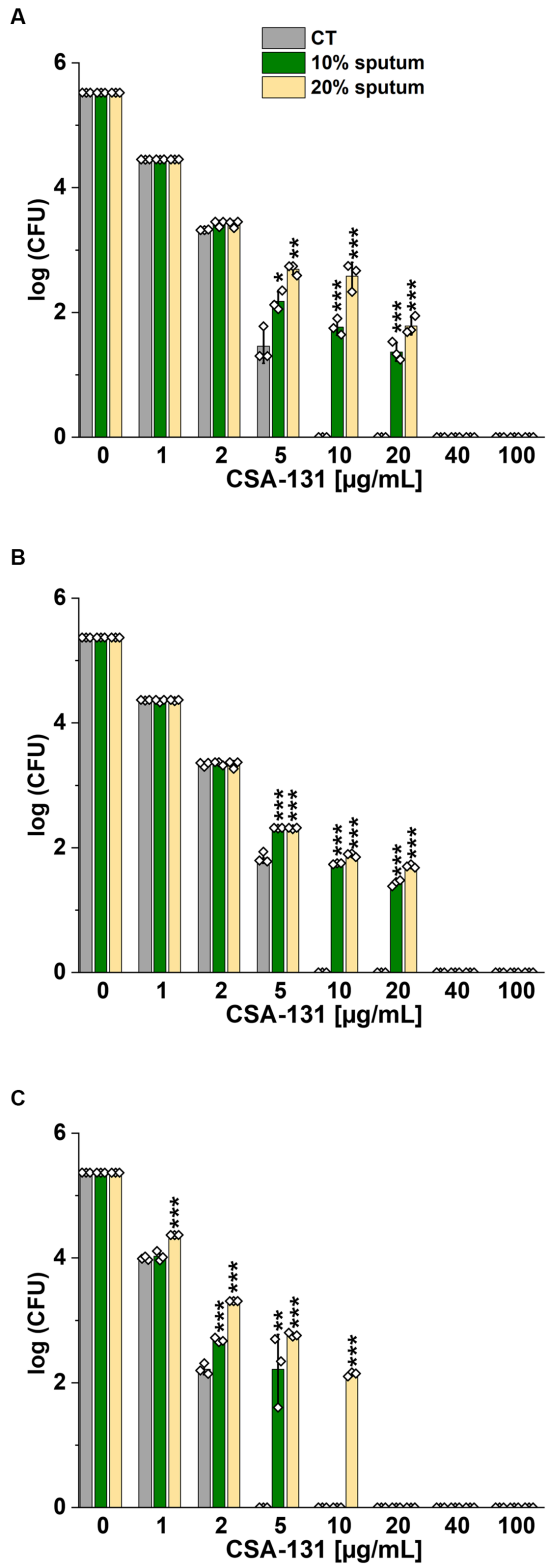
Bactericidal activity of CSA-131 against *Pseudomonas aeruginosa* ATCC 27853 **(A)** and *P. aeruginosa* PAO1 DSM 19880 **(B)** and clinical isolate *P. aeruginosa* 4B **(C)** in 10 and 20% solution of *CF* sputum collected from Patient A. Results show the mean ± SD, *n* = 3; * indicates statistical significance at *p* ≤ 0.05, ** *p* ≤ 0.01, and *** *p* ≤ 0.001 by Student’s *t*-test.

**Figure 2:**
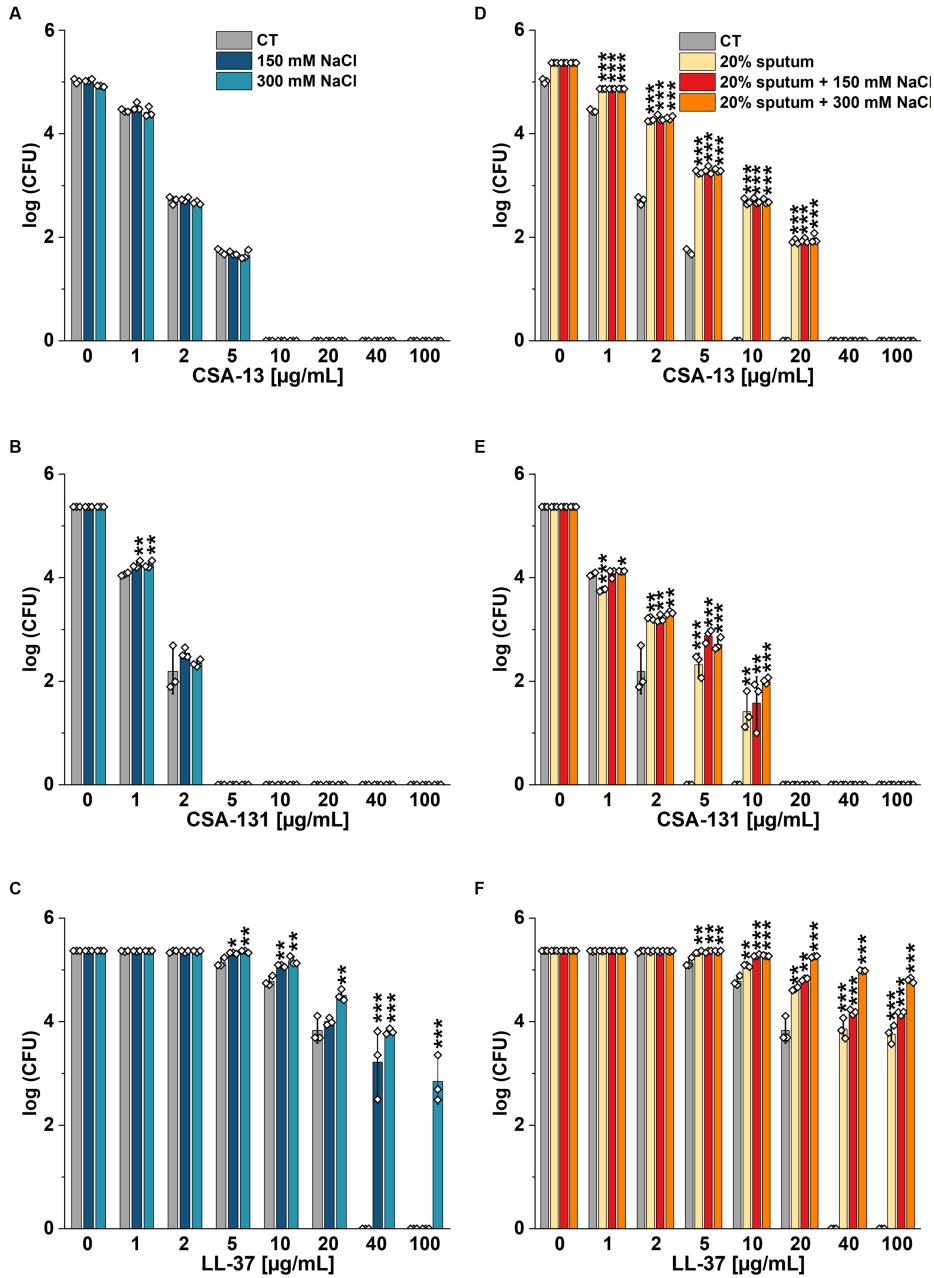
Bactericidal activity of CSA-13 **(A,D)**, CSA-131 **(B,E)**, and LL-37 **(C,F)** against *Pseudomonas aeruginosa* PAO1 DSM 19880 strain. Bacterial survival was evaluated in sterile deionized water (CT) **(A–C)**, and in 20% solution of *CF* sputum collected from Patient B **(D-F)**, without NaCl and with NaCl at a concentration of 150 mM and 300 mM. Results show the mean ± SD (*n* = 3). * indicates statistical significance at *p* ≤ 0.05, ** ≤0.01, and *** ≤0.001 by Student’s *t*-test.

**Figure 3:**
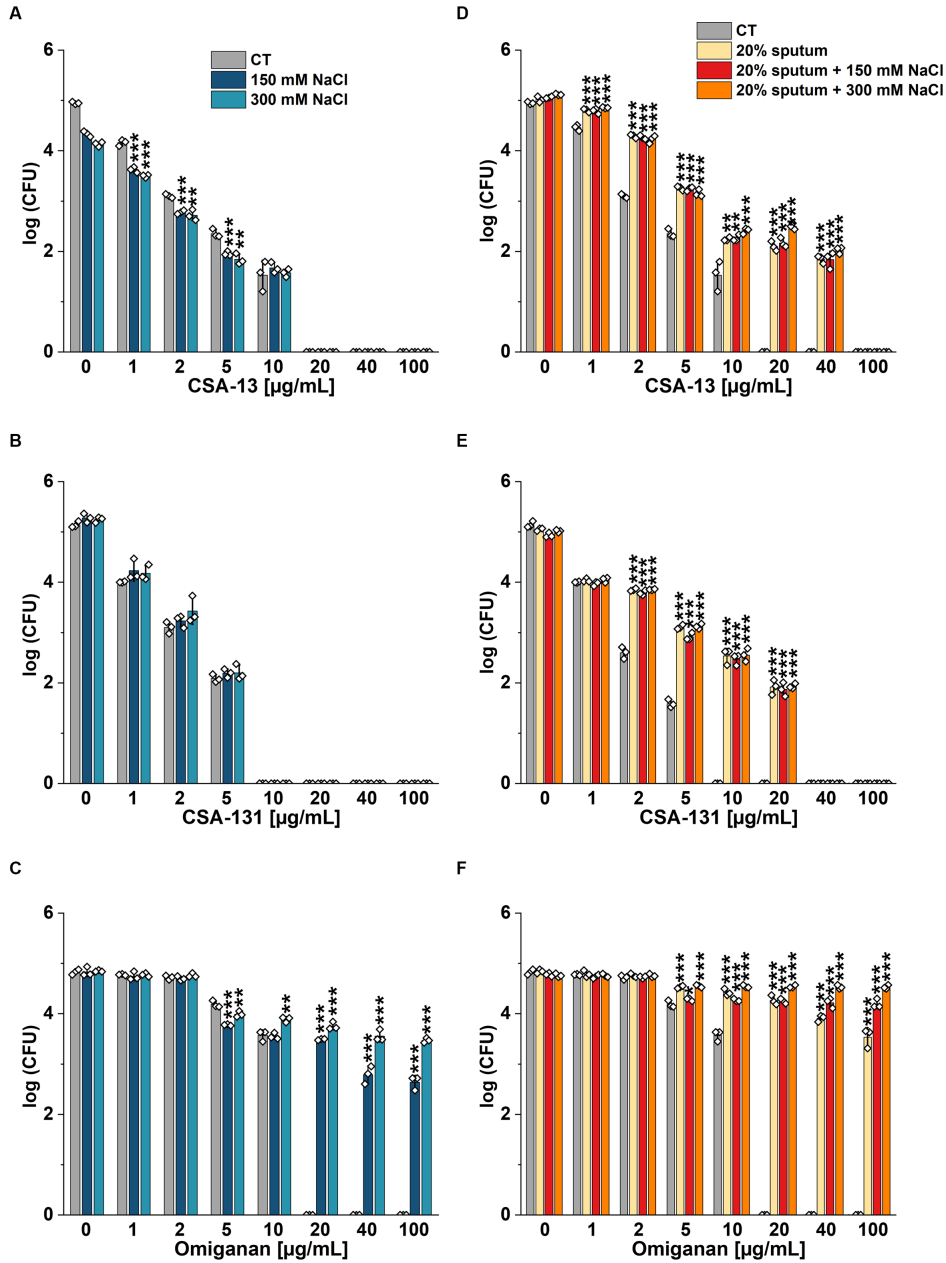
Fungicidal activity of CSA-13 **(A,D)**, CSA-131 **(B,E)**, and omiganan **(C,F)** against the clinical strain of *Candida tropicalis* 178. Fungal survival was evaluated in distilled water supplemented with NaCl **(A–C)**, and in 20% solution of *CF* sputum collected from Patient B **(D-F)** alone and supplemented with NaCl at a concentration of 150 mM and 300 mM. Results show the mean ± SD (*n* = 3). * indicates statistical significance at *p* ≤ 0.05, ** ≤0.01, and *** ≤0.001 by Student’s *t*-test.

**Figure 4:**
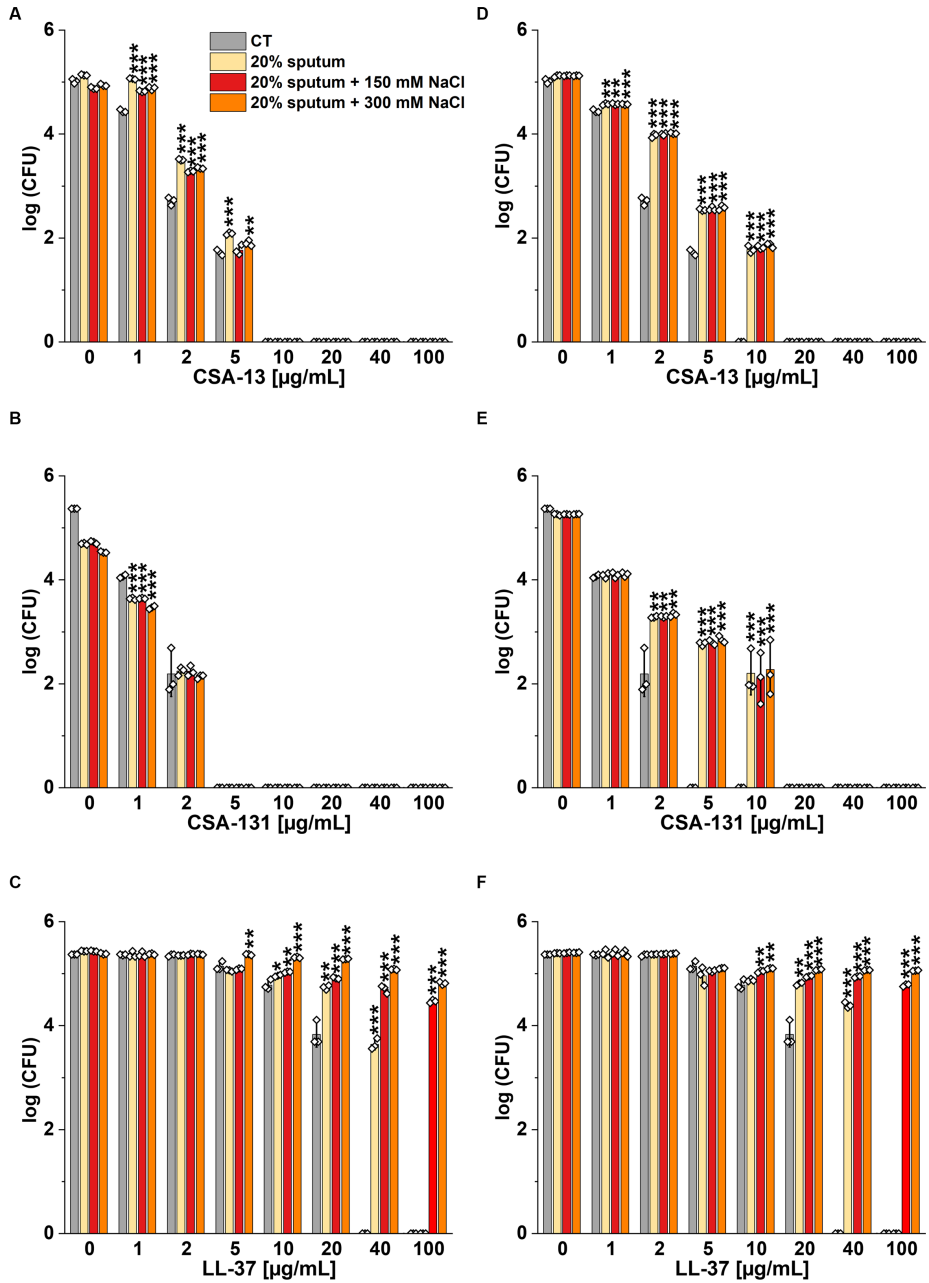
Bactericidal activity of CSA-13 **(A,D)**, CSA-131 **(B,E)**, and LL-37 **(C,F)** against *Pseudomonas aeruginosa* PAO1 DSM 19880 strain. Bacterial survival was evaluated in 20% solution of *CF* sputum collected from patient D **(A-C)**, and from Patient C **(D-F)**, without NaCl and with NaCl at a concentration of 150 mM and 300 mM. Results show the mean ± SD (*n* = 3). * indicates statistical significance at *p* ≤ 0.05, ** ≤0.01, and *** ≤0.001 by Student’s *t*-test.

**Figure 5:**
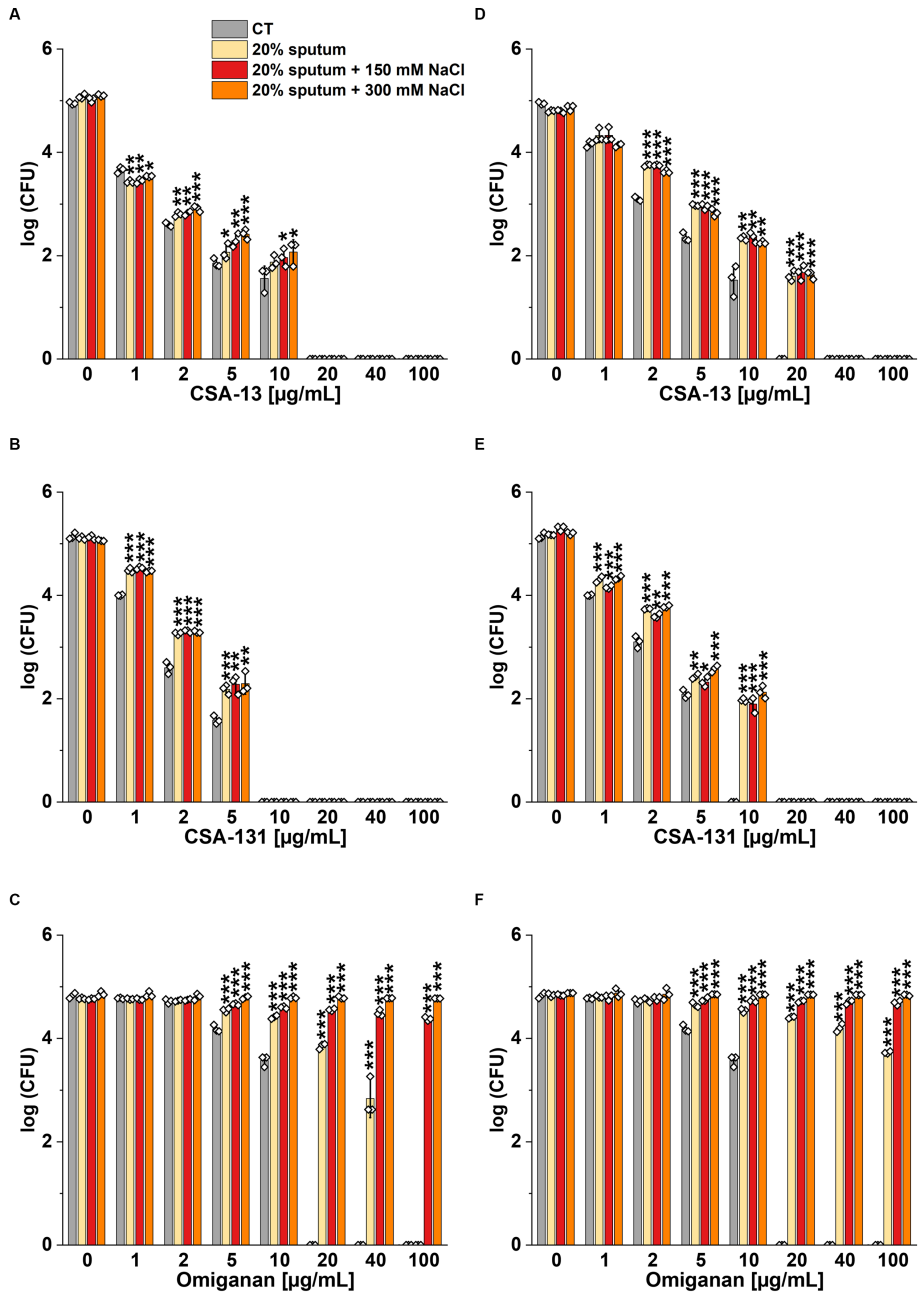
Fungicidal activity of CSA-13 **(A,D)**, CSA-131 **(B,E)**, and omiganan **(C,F)** against the clinical strain of *Candida tropicalis* 178. Fungal survival was evaluated in 20% solution of *CF* sputum from Patient D **(A-C)**, and from Patient C **(D-F)**, alone and supplemented with NaCl at a concentration of 150 mM and 300 mM. Results show the mean ± SD (*n* = 3). * indicates statistical significance at *p* ≤ 0.05, ** ≤0.01, and *** ≤0.001 by Student’s *t*-test.

The highest NaCl concentration was obtained by combining 20% sputum (containing on average 65 mM NaCl; data not shown) and 300 mM NaCl, yielding an approximate concentration of 340 mM NaCl. As shown in Figure 1, 20% sputum from Patient A decreased the antimicrobial activity of CSA-131 against all *P. aeruginosa*. In contrast, in the presence of 10% sputum, the antibacterial activity of CSA-131 against *P. aeruginosa* 1,414 remained unchanged but decreased four-fold for both *P. aeruginosa* ATCC 27853 and *P. aeruginosa* 4B. Moreover, in 20% of sputum, the antibacterial activity of CSA-131 decreased four-fold across all strains (Figure 1). Nevertheless, this inhibitory effect was not augmented by 150 mM and 300 mM NaCl (Figures 2A,B). Similar results were obtained for CSA-13, CSA-131, and *Candida tropicalis* 178 (Figures 3A,B). On the contrary, the antimicrobial activity of LL-37 and omiganan was significantly attenuated in the presence of both sputum and NaCl (Figures 2F, 3F, 4C, 4F, 5C, 5F). Briefly, NaCl alone resulted in ≥2.5-fold increase of the killing dose (concentrations at which all pathogens are effectively eradicated) for LL-37, i.e., from 40 μg/mL to over 100 μg/mL, against *P. aeruginosa* PAO1 DSM19880 strain (Figure 2C), and ≥ 5-fold increase of the killing dose for omiganan, i.e., from 20 μg/mL to over 100 μg/mL, against *C. tropicalis* 178 (Figure 3C). Furthermore, in 20% sputum from Patient B, the killing doses against *P. aeruginosa* increased from 10 to 40 μg/mL for CSA-13, and from 5 to 20 μg/mL for CSA-131 (Figures 2D,E). Similarly, the killing doses against *C. tropicalis* increased from 20 to 100 μg/mL for CSA-13 and CSA-131 (Figures 3D,E). In Patient D’s 20% sputum solution, the killing doses of both *P. aeruginosa* and *C. tropicalis* were unchanged for CSA-13 and CSA-131 (5 μg/mL) (Figures 4A,B, 5A,B), while in 20% of the sputum of Patient C, the killing doses against the test strains increased (Figures 4D,E5D,E). In addition, the presence of 20% solutions of sputum samples from each patient increased the lethal doses from 40 to 100 μg/mL for LL-37 and from 20 to 100 μg/mL for omiganan for the tested strains (Figures 2F, 3F, 4C, 4F, 5C, 5F). It should be noted that omiganan was ineffective against *C. tropicalis* in the presence of 20% sputum (regardless of the patient sample) and 300 mM (Figures 3F, 5C, 5F), while the log (CFU) for LL-37 increase from ~4.8 to ~5.05 (Figures 2F, 4C, 4F).

### 3.3 *CF* sputum contains high concentrations of DNA

**Figure 6:**
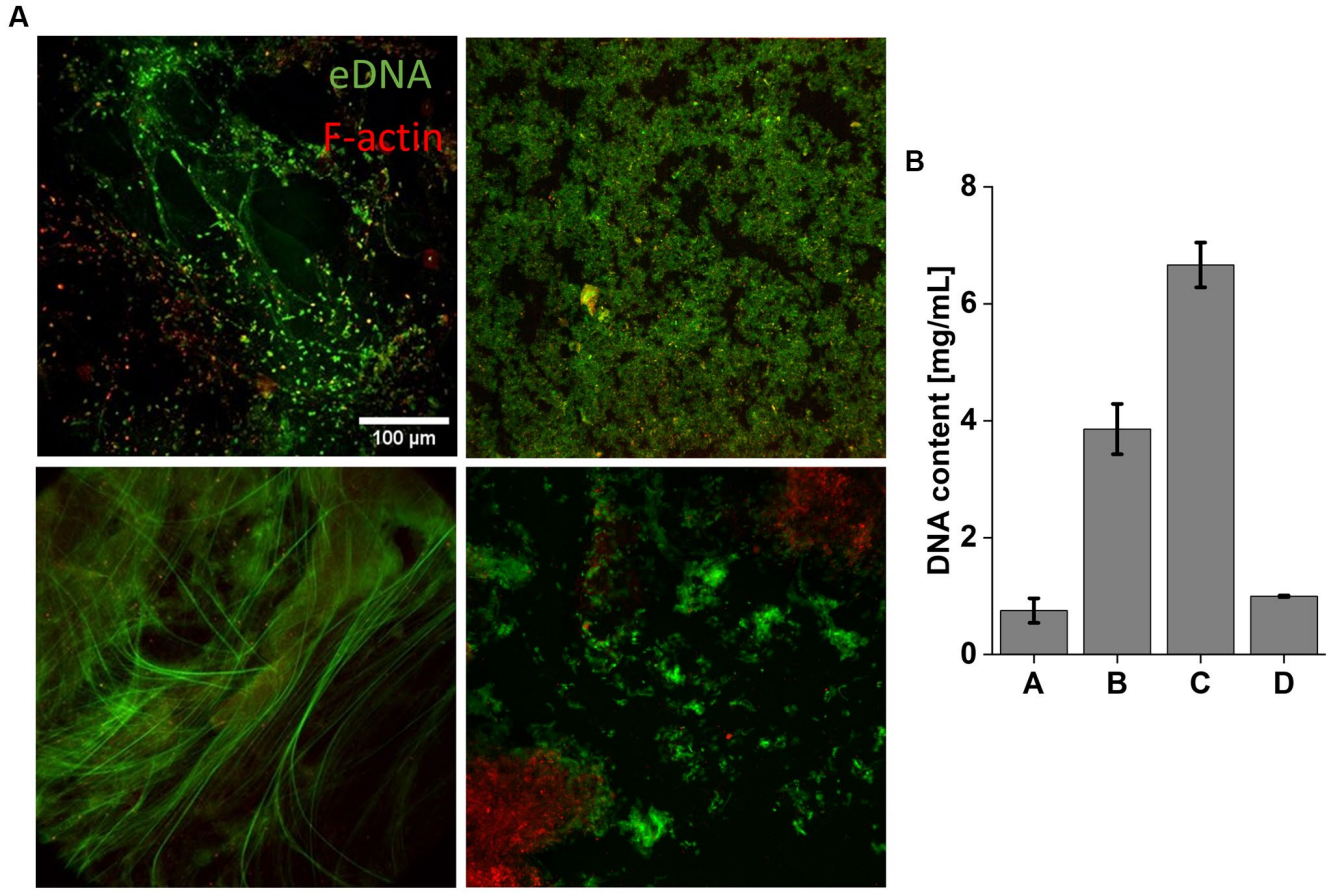
Representative microscopic images of sputum collected from *CF* patients. Top left image shows sputum from Patient A, top right image – Patient B, bottom left image – Patient C, bottom right image – Patient D. The DNA are shown green and F-actin in red. Scale bar 100 μm **(A)**. **(B)** Mean DNA concentration in *CF* sputum. Results show the mean ± SD (*n* = 3).

Among the sputum samples analyzed, the one containing *P. aeruginosa*, MRSA, and *Aspergillus* exhibited the highest DNA concentration, with a value of 6.66 ± 0.38 mg/mL. In contrast, lower DNA concentrations were observed in sputum samples containing only *P. aeruginosa* (3.86 ± 0.43 mg/mL), and the lowest DNA concentrations were found in samples with *C. albicans* (1 ± 0.01 mg/mL) and *S. aureus* (0.75 ± 0.21 mg/mL) (Figure 6B). The representative images show highly condensed bundles containing DNA and F-actin, which is characteristic of the sputum of *CF* patients (Figure 6A) (Sheils et al., 1996; Tomkiewicz et al., 1998; Tang et al., 2005).

## 4 Discussion

This study presents a comparative analysis of the antimicrobial efficacy of ceragenins and AMPs in the context of *CF* sputum and varying NaCl levels. The investigation aimed to reveal the potential of these agents as alternative therapeutic options for managing *CF*-associated infections, where conventional antibiotics frequently face limitations due to microbial resistance and the challenging *CF* microenvironment (López-Causapé et al., 2015).

Respiratory colonization and infection by *P. aeruginosa* are the leading causes of morbidity and mortality in *CF* patients. It is noteworthy that around 80% of patients are chronically colonized with *P. aeruginosa* by the age of 20 (Koch, 2002). Additionally, *CF* patients are commonly colonized by other bacterial and fungal pathogens, including methicillin-resistant *Staphylococcus aureus*, *Mycobacterium abscessus* complex, *Burkholderia cepacia* complex, *Aspergillus fumigatus*, and *Candida* species (Chotirmall et al., 2010; Bhagirath et al., 2016). Although *Ochrobactrum* infections are relatively uncommon in *CF* patients, this pathogen deserves attention due to its inherent resistance to antibiotics, the potential to cause chronic infections with clinical implications, and problems with identification (Carvalho Filho et al., 2018). *Ochrobactrum* spp. isolates have been found to exhibit high resistance to beta-lactam antibiotics, except carbapenems, due to the presence of an AmpC-like β-lactamase. Additionally, resistance to aminoglycosides and fluoroquinolones is an emerging issue that further complicates treatment efforts (Alonso et al., 2017; Yagel et al., 2020).

*CF* sputum is a complex milieu consisting of DNA, mucins, inflammatory cells, and various proteins (Morrison et al., 2019). It also contains varying levels of sodium chloride, a critical electrolyte. Studies have reported that *CF* sputum shows elevated levels of sodium chloride compared to this healthy individuals. In detail, the concentration of NaCl in the sputum of *CF* patients and healthy individuals is approximately 179.66 mM (10.5 g/L) and 126.62 mM (7.4 g/L), respectively. It is consistent with *in vivo* measurements in animal models of CFs in mice and *in vitro* in human bronchial epithelial models (Zabner et al., 1998; Jayaraman et al., 2001; Lapierre et al., 2017). Similarly, according to Joris et al., NaCl concentration in the airway surface fluid (ASF) of *CF* patients was 120–170 mM compared to 80 mM in subjects without cystic fibrosis (Joris et al., 1993). On the other hand, the average DNA concentration in *CF* sputum was found to be within the range of 0.2–20 mg/mL (White et al., 1985; Brandt et al., 1995; Lewenza, 2013; Sarkar, 2020), consistent with our findings.

The decreased antimicrobial activity of ceragenins against *P. aeruginosa* strains in the sputum may result from the presence of *S. aureus*, which was isolated from the sputum used in the study. Indeed, in the case of chronic *CF* infection, the number of microorganisms in the sputum of the respiratory tract reaches 10^7^–10^9^ CFU/mL, resulting from the inoculum effect, which substantially affects antimicrobial susceptibility testing results (Turner et al., 2015). Furthermore, while charge-based interactions can influence the antimicrobial activity of ceragenins in the presence of abundant in sputum linear polyelectrolytes, like actin or DNA, they are notably weaker compared to their impact on cationic AMPs, such as LL37, HB71, and WLBU2 (Bucki et al., 2007).

A widespread application of AMPs as antimicrobial agents is hindered by their sensitivity to several physicochemical factors frequently present in infected sites, such as a high salt concentration in the *CF* airway surface fluid (Goldman et al., 1997). For instance, in patients with cystic fibrosis, the activity of AMPs is significantly lower in the ASL than in normal ASL (Smith et al., 1996; Hiemstra, 2007).

Indeed, our results show that the antimicrobial activity of both LL-37 and omiganan against *CF*-associated pathogens is NaCl concentration-dependent. Similarly, Bals et al. and Tanaka et al. demonstrated that the antimicrobial activity of LL-37 diminishes with rising NaCl concentrations (Bals et al., 1998; Tanaka et al., 2000). Several studies have highlighted the limitations of AMPs in their antimicrobial activity. A high-salt environment has been found to impair the effectiveness of AMPs, as observed in the case of peptides like indolicidin, human beta-defensin-1, histidine-rich peptide P-113, gramicidins, magainins, and bactenecins (Goldman et al., 1997; Lee et al., 1997; Wu et al., 1999; Rothstein et al., 2001). In *CF* sputum, the presence of bacterial endotoxin lipopolysaccharide (LPS) is one of the factors leading to the inhibition of LL-37 antimicrobial activity (Bucki et al., 2007). Additionally, the secretion of two zinc-dependent metalloproteases, ZmpA and ZmpB, by *Burkholderia cepacia* can lead to the inactivation of AMPs, such as human beta-defensin-1 and LL-37 (Kooi and Sokol, 2009). These findings shed light on the challenges in utilizing AMPs as effective therapeutic agents in *CF* patients.

The structure of an AMP plays a vital role in determining its antimicrobial activity. It is believed that the helical, oligomeric conformation of LL-37 is crucial to the protein’s antimicrobial activity. Hence, the highest LL-37 antimicrobial activity is observed with maximum helix content, while intermediate and low activity corresponds to lower helix content and a disordered secondary structure. These findings suggest that the optimal antimicrobial activity of LL-37 necessitates an oligomeric α-helical structure before its interaction with the bacterial membrane (Johansson et al., 1998). Studies have demonstrated that increased NaCl concentration can induce conformational changes in LL-37. Specifically, at higher NaCl concentrations, LL-37 may undergo structural alterations and adopt a more random or disordered conformation resulting in decreased antimicrobial activity (Park et al., 2004; Huang et al., 2014).

In contrast, omiganan does not display a helical structure. However, the presence of positively charged sodium ions affects electrostatic interactions of this cationic AMP with microbial cell membranes (Mojsoska and Jenssen, 2015; Ghosh et al., 2021). Hence, in high-salt environments, the antimicrobial activity of omiganan may decrease, rendering it less effective against certain microorganisms. This dependence on salt concentration can affect its efficacy in physiological conditions where salt levels fluctuate, such as respiratory tract or skin infections (Brown, 2021).

On the other hand, the antimicrobial activity of ceragenins is independent of the concentration of NaCl, likely due to their unique structural properties. The smaller size of CSA-13 and CSA-131 than LL-37 and omiganan, along with distinct charge densities and lower positive charges, ensure their stability and resistance to changes in the ionic environment, including fluctuations in NaCl concentration and anionic polyelectrolytes, such as extracellular DNA (eDNA), F-actin, and mucin (Hashemi et al., 2018). In addition, the direct targeting of membrane lipids by ceragenins makes them less vulnerable to salt concentration compared to the highly charge-based AMPs mechanism of action (Epand et al., 2007).

Mucoid strains of *P. aeruginosa* that produce a thick alginate biofilm are typically more resistant to antimicrobials than non-mucoid strains due to the protective nature of the biofilm, which limits their penetration and, consequently, their efficacy (Nichols et al., 1989; Meluleni et al., 1995; Stewart, 1996; Hentzer et al., 2001). *P. aeruginosa* is well-known for its ability to develop antibiotic resistance, for example, through upregulation of genes encoding efflux pumps, which effectively remove antibiotics from the cell (Tomás et al., 2010). The favorable biocompatibility of CSA has been reported in previous work (Piktel et al., 2020; Paprocka et al., 2021; Suprewicz et al., 2023). For CSA-13 and CSA-131, the viability of human basal alveolar epithelial adenocarcinoma cells (A549) at a concentration of 10 μg/mL CSA-13 and CSA-131 was maintained at approximately 60 and 80%, respectively (Piktel et al., 2020; Paprocka et al., 2021; Suprewicz et al., 2023). Additionally, the problem of cytotoxicity can be effectively solved by the incorporation of Pluronic, a compound that increases cell viability and alleviates the hemolytic effects of CSAs while maintaining their antimicrobial activity (Leszczyńska et al., 2011; Paprocka et al., 2021).

Understanding the disparate modes of action and resistance to changes in salt concentration provides valuable insights into the therapeutic potential of ceragenins as effective antimicrobial agents, particularly in managing infections in complex biological environments, such as *CF* lungs. Further research in this area is critical to elucidate the intricacies of ceragenin activity and its implications for clinical applications. The intrinsic stability and resistance to changes in NaCl concentration make ceragenins promising candidates for combating infections in complex environments like the *CF* sputum (Bucki et al., 2007; Leszczyńska et al., 2011).

## 5 Conclusion

Our study provides a comprehensive comparative analysis of the efficacy of ceragenins and AMPs in the context of *CF* sputum and varying NaCl concentrations. We investigated the antimicrobial activity of CSA-13, CSA-131, LL-37, and omiganan against *CF*-associated pathogens, evaluating their performance under different conditions. Our findings revealed that ceragenins, specifically CSA-13 and CSA-131, exhibited remarkable antimicrobial efficacy, surpassing natural (LL-37) and synthetic (omiganan) AMPs. Notably, the antimicrobial activity of ceragenins remained consistent across a wide range of NaCl concentrations, underscoring their resistance to changes in ionic environments. In contrast, the antimicrobial activity of LL-37 and omiganan was reduced in high-salt environments, potentially compromising their performance in *CF* sputum, a condition where NaCl levels can vary. The structural properties of ceragenins and their lipid-based mode of action likely contribute to their stability and sustained antimicrobial activity, even under salt excess condition. These properties highlight the potential of ceragenins as promising candidates for combating *CF*-associated infections and overcoming limitations observed in natural and synthetic AMPs.

## Scope statement

The study investigated the effect of sodium chloride (NaCl) on the antimicrobial efficacy of the cathelicidin LL-37, and their synthetic mimetic ceragenins (Ceragenins), which are considered as potential therapeutic agents in cystic fibrosis (*CF*) lung infections. The susceptibility of cystic fibrosis-associated bacterial pathogens (*Pseudomonas aeruginosa*, *Ochrobactrum* spp. and *Staphylococcus aureus*) and fungal pathogens (*Candida albicans* and *C. tropicalis*) to these compounds was determined using Minimum Inhibitory Concentrations (MIC) and Bactericidal Concentrations (MBC) tests in sputum samples collected from patients diagnosed with *CF*. Furthermore, a colony-counting assay was used to assess the effect of varying NaCl concentrations on tested agents’ antimicrobial activity. The findings reveal that ceragenins exhibit potent antimicrobial activity in *CF* sputum, regardless of the NaCl concentration when compared to LL-37 and omiganan. Given the broad-spectrum antimicrobial activity of ceragenins in microenvironments resembling the airways of *CF* patients, they represent promising agents for managing *CF* disease. This manuscript aligns with the journal’s scope as it contributes to the field of microbiology and infectious diseases. The investigation provides valuable insights into the development of effective antimicrobial strategies to combat *CF*-associated infections.

## Data availability statement

The raw data supporting the conclusions of this article will be made available by the authors, without undue reservation.

## Ethics statement

The studies involving humans were approved by Bioethics Committee of Medical University of Bialystok. The studies were conducted in accordance with the local legislation and institutional requirements. The participants provided their written informed consent to participate in this study.

## Author contributions

KS: Conceptualization, Formal analysis, Writing – original draft, Data curation, Investigation, Methodology, Software, Writing – review & editing. ŁS: Formal analysis, Investigation, Methodology, Software, Writing – review & editing, Visualization. SC-D: Methodology, Software, Visualization, Writing – review & editing. SK: Methodology, Writing – review & editing, Investigation, Resources. SO: Methodology, Writing – review & editing, Software, Visualization. MZ: Software, Formal analysis, Investigation, Writing – original draft. ŁM: Formal analysis, Investigation, Data curation, Resources, Writing – review & editing. RM: Formal analysis, Investigation, Resources, Writing – review & editing, Supervision. TD: Formal analysis, Investigation, Writing – review & editing, Methodology, Validation. PS: Formal analysis, Investigation, Validation, Writing – review & editing, Supervision. KF: Formal analysis, Supervision, Validation, Writing – review & editing, Project administration, Visualization, Writing – original draft. RB: Formal analysis, Validation, Writing – original draft, Conceptualization, Resources.

## References

[ref1] Akkerman-Nijland A. M. Akkerman O. W. Grasmeijer F. Hagedoorn P. Frijlink H. W. Rottier B. L.. (2021). The pharmacokinetics of antibiotics in cystic fibrosis. Expert Opin. Drug Metab. Toxicol. 17, 53–68. doi: 10.1080/17425255.2021.1836157 33213220

[ref2] Alonso C. A. Kwabugge Y. A. Anyanwu M. U. Torres C. Chah K. F. (2017). Diversity of Ochrobactrum species in food animals, antibiotic resistance phenotypes and polymorphisms in the bla OCH gene. FEMS Microbiol. Lett. 364:178. doi: 10.1093/femsle/fnx178 28911188

[ref3] Anderson R. C. Yu P.-L. (2005). Factors affecting the antimicrobial activity of ovine-derived cathelicidins against *E. Coli* 0157: H7. Int. J. Antimicrob. Agents 25, 205–210. doi: 10.1016/j.ijantimicag.2004.10.010, PMID: 15737513

[ref4] Bals R. Wang X. Zasloff M. Wilson J. M. (1998). The peptide antibiotic LL-37/hCAP-18 is expressed in epithelia of the human lung where it has broad antimicrobial activity at the airway surface. Proc. Natl. Acad. Sci. 95, 9541–9546. doi: 10.1073/pnas.95.16.9541, PMID: 9689116 PMC21374

[ref5] Bhagirath A. Y. Li Y. Somayajula D. Dadashi M. Badr S. Duan K. (2016). Cystic fibrosis lung environment and *Pseudomonas aeruginosa* infection. BMC Pulm. Med. 16:174. doi: 10.1186/s12890-016-0339-5, PMID: 27919253 PMC5139081

[ref6] Bhat P. G. Flanagan D. R. Donovan M. D. (1996). Drug diffusion through cystic fibrotic mucus: steady-state permeation, rheologic properties, and glycoprotein morphology. J. Pharm. Sci. 85, 624–630. doi: 10.1021/js950381s, PMID: 8773960

[ref7] Boucher R. (2004). New concepts of the pathogenesis of cystic fibrosis lung disease. Eur. Respir. J. 23, 146–158. doi: 10.1183/09031936.03.00057003, PMID: 14738247

[ref8] Brandt T. Breitenstein S. von der Hardt H. Tümmler B. (1995). DNA concentration and length in sputum of patients with cystic fibrosis during inhalation with recombinant human DNase. Thorax 50, 880–882. doi: 10.1136/thx.50.8.880, PMID: 7570441 PMC474911

[ref9] Brown R. B. (2021). Sodium toxicity in the nutritional epidemiology and nutritional immunology of COVID-19. Medicina 57:739. doi: 10.3390/medicina57080739, PMID: 34440945 PMC8399536

[ref10] Bucki R. Byfield F. J. Janmey P. A. (2007). Release of the antimicrobial peptide LL-37 from DNA/F-actin bundles in cystic fibrosis sputum. Eur. Respir. J. 29, 624–632. doi: 10.1183/09031936.00080806, PMID: 17215317

[ref11] Bucki R. Niemirowicz K. Wnorowska U. Byfield F. J. Piktel E. Wątek M.. (2015). Bactericidal activity of ceragenin CSA-13 in cell culture and in an animal model of peritoneal infection. Antimicrob. Agents Chemother. 59, 6274–6282. doi: 10.1128/AAC.00653-15, PMID: 26248361 PMC4576122

[ref12] Bucki R. Sostarecz A. G. Byfield F. J. Savage P. B. Janmey P. A. (2007). Resistance of the antibacterial agent ceragenin CSA-13 to inactivation by DNA or F-actin and its activity in cystic fibrosis sputum. J. Antimicrob. Chemother. 60, 535–545. doi: 10.1093/jac/dkm218, PMID: 17584802

[ref13] Carvalho Filho É. B. Marson F. A. L. Levy C. E. (2018). Challenges in the identification of *Ochrobactrum anthropi* in blood and sputum cultures of patients with cystic fibrosis. Rev. Epidemiol. E Controle Infecção. 8, 189–191. doi: 10.17058/reci.v1i2.9967

[ref14] Chmielewska S. J. Skłodowski K. Piktel E. Suprewicz Ł. Fiedoruk K. Daniluk T.. (2020). NDM-1 Carbapenemase-producing Enterobacteriaceae are highly susceptible to Ceragenins CSA-13, CSA-44, and CSA-131. Infect. Drug Resist. 13, 3277–3294. doi: 10.2147/IDR.S261579, PMID: 33061475 PMC7535143

[ref15] Choi K.-H. Kumar A. Schweizer H. P. (2006). A 10-min method for preparation of highly electrocompetent *Pseudomonas aeruginosa* cells: application for DNA fragment transfer between chromosomes and plasmid transformation. J. Microbiol. Methods 64, 391–397. doi: 10.1016/j.mimet.2005.06.001, PMID: 15987659

[ref16] Chotirmall S. H. Greene C. M. McElvaney N. G. (2010). Candida species in cystic fibrosis: a road less travelled. Med. Mycol. 48, S114–S124. doi: 10.3109/13693786.2010.503320 21067323

[ref17] Dagenais R. V. Su V. C. Quon B. S. (2020). Real-world safety of CFTR modulators in the treatment of cystic fibrosis: a systematic review. J. Clin. Med. 10:23. doi: 10.3390/jcm10010023, PMID: 33374882 PMC7795777

[ref18] Ding B. Guan Q. Walsh J. P. Boswell J. S. Winter T. W. Winter E. S.. (2002). Correlation of the antibacterial activities of cationic peptide antibiotics and cationic steroid antibiotics. J. Med. Chem. 45, 663–669. doi: 10.1021/jm0105070, PMID: 11806717

[ref19] Durnaś B. Piktel E. Wątek M. Wollny T. Góźdź S. Smok-Kalwat J.. (2017). Anaerobic bacteria growth in the presence of cathelicidin LL-37 and selected ceragenins delivered as magnetic nanoparticles cargo. BMC Microbiol. 17:167. doi: 10.1186/s12866-017-1075-6, PMID: 28747178 PMC5530502

[ref20] Epand R. F. Savage P. B. Epand R. M. (2007). Bacterial lipid composition and the antimicrobial efficacy of cationic steroid compounds (Ceragenins). Biochim. Biophys. Acta 1768, 2500–2509. doi: 10.1016/j.bbamem.2007.05.023, PMID: 17599802

[ref21] Felgentreff K. Beisswenger C. Griese M. Gulder T. Bringmann G. Bals R. (2006). The antimicrobial peptide cathelicidin interacts with airway mucus. Peptides 27, 3100–3106. doi: 10.1016/j.peptides.2006.07.018, PMID: 16963160

[ref22] Ghosh S. Pandit G. Debnath S. Chatterjee S. Satpati P. (2021). Effect of monovalent salt concentration and peptide secondary structure in peptide-micelle binding. RSC Adv. 11, 36836–36849. doi: 10.1039/D1RA06772A, PMID: 35494385 PMC9043568

[ref23] Goldman M. J. Anderson G. M. Stolzenberg E. D. Kari U. P. Zasloff M. Wilson J. M. (1997). Human β-defensin-1 is a salt-sensitive antibiotic in lung that is inactivated in cystic fibrosis. Cells 88, 553–560. doi: 10.1016/S0092-8674(00)81895-4, PMID: 9038346

[ref24] Habib A.-R. R. Kajbafzadeh M. Desai S. Yang C. L. Skolnik K. Quon B. S. (2019). A systematic review of the clinical efficacy and safety of CFTR modulators in cystic fibrosis. Sci. Rep. 9:7234. doi: 10.1038/s41598-019-43652-2, PMID: 31076617 PMC6510767

[ref25] Haq I. Almulhem M. Soars S. Poulton D. Brodlie M. (2022). Precision medicine based on CFTR genotype for people with cystic fibrosis. Pharmgenomics Pers. Med. 15, 91–104. doi: 10.2147/PGPM.S245603, PMID: 35153502 PMC8828078

[ref26] Hashemi M. M. Holden B. S. Savage P. B. (2018). “Ceragenins as non-peptide mimics of endogenous antimicrobial peptides” in Fighting Antimicrobial Resistance. ed. Budimir A. (Zagreb, Croatia: IAPC Publishing), 139–169.

[ref27] Hentzer M. Teitzel G. M. Balzer G. J. Heydorn A. Molin S. Givskov M.. (2001). Alginate overproduction affects *Pseudomonas aeruginosa* biofilm structure and function. J. Bacteriol. 183, 5395–5401. doi: 10.1128/JB.183.18.5395-5401.2001, PMID: 11514525 PMC95424

[ref28] Hiemstra P. (2007). Antimicrobial peptides in the real world: implications for cystic fibrosis. Eur. Respir. J. 29, 617–618. doi: 10.1183/09031936.00017007, PMID: 17400874

[ref29] Howell M. D. Streib J. E. Kim B. E. Lesley L. J. Dunlap A. P. Geng D.. (2009). Ceragenins: a class of antiviral compounds to treat orthopox infections. J. Investig. Dermatol. 129, 2668–2675. doi: 10.1038/jid.2009.120, PMID: 19516269 PMC8609773

[ref30] Huang Y. He L. Li G. Zhai N. Jiang H. Chen Y. (2014). Role of helicity of α-helical antimicrobial peptides to improve specificity. Protein Cell 5, 631–642. doi: 10.1007/s13238-014-0061-0, PMID: 24805306 PMC4130925

[ref31] Javia A. Misra A. Thakkar H. (2022). Liposomes encapsulating novel antimicrobial peptide Omiganan: characterization and its pharmacodynamic evaluation in atopic dermatitis and psoriasis mice model. Int. J. Pharm. 624:122045. doi: 10.1016/j.ijpharm.2022.122045, PMID: 35878872

[ref32] Jayaraman S. Song Y. Vetrivel L. Shankar L. Verkman A. (2001). Noninvasive in vivo fluorescence measurement of airway-surface liquid depth, salt concentration, and pH. J. Clin. Invest. 107, 317–324. doi: 10.1172/JCI11154, PMID: 11160155 PMC199195

[ref33] Johansson J. Gudmundsson G. H. MnE R. Berndt K. D. Agerberth B. (1998). Conformation-dependent antibacterial activity of the naturally occurring human peptide LL-37. J. Biol. Chem. 273, 3718–3724. doi: 10.1074/jbc.273.6.3718, PMID: 9452503

[ref34] Joris L. Dab I. Quinton P. M. (1993). Elemental composition of human airway surface fluid in healthy and diseased airways. Am. J. Respir. Crit. Care Med. 148, 1633–1637. doi: 10.1164/ajrccm/148.6_Pt_1.1633 8256912

[ref35] Kandasamy S. K. Larson R. G. (2006). Effect of salt on the interactions of antimicrobial peptides with zwitterionic lipid bilayers. Biochim. Biophys. Acta 1758, 1274–1284. doi: 10.1016/j.bbamem.2006.02.030, PMID: 16603122

[ref36] Koch C. (2002). Early infection and progression of cystic fibrosis lung disease. Pediatr. Pulmonol. 34, 232–236. doi: 10.1002/ppul.10135 12203855

[ref37] Kooi C. Sokol P. A. (2009). *Burkholderia cenocepacia* zinc metalloproteases influence resistance to antimicrobial peptides. Microbiology 155, 2818–2825. doi: 10.1099/mic.0.028969-0, PMID: 19542010

[ref38] Lapierre S. G. Phelippeau M. Hakimi C. Didier Q. Reynaud-Gaubert M. Dubus J.-C. (2017). Cystic fibrosis respiratory tract salt concentration: an exploratory cohort study. Medicine 96:e8423. doi: 10.1097/MD.0000000000008423 29381919 PMC5708918

[ref39] Lee I. H. Cho Y. Lehrer R. I. (1997). Effects of pH and salinity on the antimicrobial properties of clavanins. Infect. Immun. 65, 2898–2903. doi: 10.1128/iai.65.7.2898-2903.1997, PMID: 9199465 PMC175407

[ref40] Lei J. Sun L. Huang S. Zhu C. Li P. He J.. (2019). The antimicrobial peptides and their potential clinical applications. Am. J. Transl. Res. 11, 3919–3931. PMID: 31396309 PMC6684887

[ref41] Leszczyńska K. Namiot A. Cruz K. Byfield F. Won E. Mendez G. (2011). Potential of ceragenin CSA-13 and its mixture with pluronic F-127 as treatment of topical bacterial infections. J. Appl. Microbiol. 110, 229–238. doi: 10.1111/j.1365-2672.2010.04874.x, PMID: 20961363 PMC3386848

[ref42] Lewenza S. (2013). Extracellular DNA-induced antimicrobial peptide resistance mechanisms in *Pseudomonas aeruginosa*. Front. Microbiol. 4:21. doi: 10.3389/fmicb.2013.00021 23419933 PMC3572637

[ref43] López-Causapé C. Rojo-Molinero E. Macia M. D. Oliver A. (2015). The problems of antibiotic resistance in cystic fibrosis and solutions. Expert Rev. Respir. Med. 9, 73–88. doi: 10.1586/17476348.2015.995640, PMID: 25541089

[ref44] Lyczak J. B. Cannon C. L. Pier G. B. (2002). Lung infections associated with cystic fibrosis. Clin. Microbiol. Rev. 15, 194–222. doi: 10.1128/CMR.15.2.194-222.2002, PMID: 11932230 PMC118069

[ref45] Majewski K. Kozłowska E. Żelechowska P. Brzezińska-Błaszczyk E. (2018). Serum concentrations of antimicrobial peptide cathelicidin LL-37 in patients with bacterial lung infections. Cent. Eur. J. Immunol. 43, 453–457. doi: 10.5114/ceji.2018.81355, PMID: 30799994 PMC6384432

[ref46] Mansour S. C. Pena O. M. Hancock R. E. (2014). Host defense peptides: front-line immunomodulators. Trends Immunol. 35, 443–450. doi: 10.1016/j.it.2014.07.004, PMID: 25113635

[ref47] McGarry M. E. Gibb E. R. Oates G. R. Schechter M. S. (2022). Left behind: the potential impact of CFTR modulators on racial and ethnic disparities in cystic fibrosis. Paediatr. Respir. Rev. 42, 35–42. doi: 10.1016/j.prrv.2021.12.001, PMID: 35277357 PMC9356388

[ref48] McKelvey M. C. Weldon S. McAuley D. F. Mall M. A. Taggart C. C. (2020). Targeting proteases in cystic fibrosis lung disease. Paradigms, progress, and potential. Am. J. Respir. Crit. Care Med. 201, 141–147. doi: 10.1164/rccm.201906-1190PP, PMID: 31626562 PMC6961750

[ref49] Meluleni G. J. Grout M. Evans D. J. Pier G. B. (1995). Mucoid *Pseudomonas aeruginosa* growing in a biofilm in vitro are killed by opsonic antibodies to the mucoid exopolysaccharide capsule but not by antibodies produced during chronic lung infection in cystic fibrosis patients. J. Immunol. 155, 2029–2038. doi: 10.4049/jimmunol.155.4.2029, PMID: 7636254

[ref50] Mojsoska B. Jenssen H. (2015). Peptides and peptidomimetics for antimicrobial drug design. Pharmaceuticals 8, 366–415. doi: 10.3390/ph8030366, PMID: 26184232 PMC4588174

[ref51] Morrison C. B. Markovetz M. R. Ehre C. (2019). Mucus, mucins, and cystic fibrosis. Pediatr. Pulmonol. 54, S84–S96. doi: 10.1002/ppul.24530, PMID: 31715083 PMC6853602

[ref52] Ng S. M. S. Teo S. W. Yong Y. E. Ng F. M. Lau Q. Y. Jureen R.. (2017). Preliminary investigations into developing all-D Omiganan for treating mupirocin-resistant MRSA skin infections. Chem. Biol. Drug Des. 90, 1155–1160. doi: 10.1111/cbdd.13035, PMID: 28581672

[ref53] Nichols W. W. Evans M. J. Slack M. P. Walmsley H. L. (1989). The penetration of antibiotics into aggregates of mucoid and non-mucoid *Pseudomonas aeruginosa*. Microbiology 135, 1291–1303. doi: 10.1099/00221287-135-5-1291, PMID: 2516117

[ref54] Nivens D. E. Ohman D. E. Williams J. Franklin M. J. (2001). Role of alginate and its O acetylation in formation of *Pseudomonas aeruginosa* microcolonies and biofilms. J. Bacteriol. 183, 1047–1057. doi: 10.1128/JB.183.3.1047-1057.2001, PMID: 11208804 PMC94973

[ref55] Paprocka P. Durnaś B. Mańkowska A. Skłodowski K. Król G. Zakrzewska M.. (2021). New β-lactam antibiotics and ceragenins–a study to assess their potential in treatment of infections caused by multidrug-resistant strains of *Pseudomonas aeruginosa*. Infect. Drug Resist. 14, 5681–5698. doi: 10.2147/IDR.S338827, PMID: 34992394 PMC8715797

[ref56] Paprocka P. Mańkowska A. Skłodowski K. Król G. Wollny T. Lesiak A.. (2022). Bactericidal activity of Ceragenin in combination with ceftazidime, levofloxacin, co-Trimoxazole, and Colistin against the opportunistic pathogen *Stenotrophomonas maltophilia*. Pathogens 11:621. doi: 10.3390/pathogens11060621, PMID: 35745475 PMC9227598

[ref57] Park I. Y. Cho J. H. Kim K. S. Kim Y.-B. Kim M. S. Kim S. C. (2004). Helix stability confers salt resistance upon helical antimicrobial peptides. J. Biol. Chem. 279, 13896–13901. doi: 10.1074/jbc.M311418200, PMID: 14718539

[ref58] Piktel E. Markiewicz K. H. Wilczewska A. Z. Daniluk T. Chmielewska S. Niemirowicz-Laskowska K.. (2020). Quantification of synergistic effects of Ceragenin CSA-131 combined with iron oxide magnetic nanoparticles against cancer cells. Int. J. Nanomedicine 15, 4573–4589. doi: 10.2147/IJN.S255170, PMID: 32606693 PMC7321689

[ref59] Pollard J. E. Snarr J. Chaudhary V. Jennings J. D. Shaw H. Christiansen B.. (2012). In vitro evaluation of the potential for resistance development to ceragenin CSA-13. J. Antimicrob. Chemother. 67, 2665–2672. doi: 10.1093/jac/dks276, PMID: 22899801 PMC3468081

[ref60] Pragman A. A. Berger J. P. Williams B. J. (2016). Understanding persistent bacterial lung infections: clinical implications informed by the biology of the microbiota and biofilms. Clin. Pulm. Med. 23, 57–66. doi: 10.1097/CPM.0000000000000108, PMID: 27004018 PMC4798234

[ref61] Rossi L. M. Rangasamy P. Zhang J. Qiu X. Q. Wu G. Y. (2008). Research advances in the development of peptide antibiotics. J. Pharm. Sci. 97, 1060–1070. doi: 10.1002/jps.21053 17694545

[ref62] Rothstein D. M. Spacciapoli P. Tran L. T. Xu T. Roberts F. D. Dalla Serra M.. (2001). Anticandida activity is retained in P-113, a 12-amino-acid fragment of histatin 5. Antimicrob. Agents Chemother. 45, 1367–1373. doi: 10.1128/AAC.45.5.1367-1373.2001, PMID: 11302797 PMC90475

[ref63] Saint-Criq V. Gray M. A. (2017). Role of CFTR in epithelial physiology. Cell. Mol. Life Sci. 74, 93–115. doi: 10.1007/s00018-016-2391-y, PMID: 27714410 PMC5209439

[ref64] Sarkar S. (2020). Release mechanisms and molecular interactions of *Pseudomonas aeruginosa* extracellular DNA. Appl. Microbiol. Biotechnol. 104, 6549–6564. doi: 10.1007/s00253-020-10687-9, PMID: 32500267

[ref65] Schaupp L. Addante A. Völler M. Fentker K. Kuppe A. Bardua M.. (2023). Longitudinal effects of elexacaftor/tezacaftor/ivacaftor on sputum viscoelastic properties, airway infection and inflammation in patients with cystic fibrosis. Eur. Respir. J. 62:2202153. doi: 10.1183/13993003.02153-2022, PMID: 37414422

[ref66] Sheils C. A. Käs J. Travassos W. Allen P. G. Janmey P. A. Wohl M. E.. (1996). Actin filaments mediate DNA fiber formation in chronic inflammatory airway disease. Am. J. Pathol. 148, 919–927. PMID: 8774146 PMC1861734

[ref67] Simonin J. Bille E. Crambert G. Noel S. Dreano E. Edwards A.. (2019). Author correction: airway surface liquid acidification initiates host defense abnormalities in cystic fibrosis. Sci. Rep. 9:17535. doi: 10.1038/s41598-019-54253-4, PMID: 31754179 PMC6872590

[ref68] Sinclair K. Pham T. Farnsworth R. Williams D. Loc-Carrillo C. Horne L.. (2012). Development of a broad spectrum polymer-released antimicrobial coating for the prevention of resistant strain bacterial infections. J. Biomed. Mater. Res. A 100, 2732–2738. doi: 10.1002/jbm.a.34209, PMID: 22623404 PMC3429640

[ref69] Smith J. J. Travis S. M. Greenberg E. P. Welsh M. J. (1996). Cystic fibrosis airway epithelia fail to kill bacteria because of abnormal airway surface fluid. Cells 85, 229–236. doi: 10.1016/S0092-8674(00)81099-5, PMID: 8612275

[ref70] Stewart P. S. (1996). Theoretical aspects of antibiotic diffusion into microbial biofilms. Antimicrob. Agents Chemother. 40, 2517–2522. doi: 10.1128/AAC.40.11.2517, PMID: 8913456 PMC163567

[ref71] Suprewicz Ł. Szczepański A. Lenart M. Piktel E. Fiedoruk K. Barreto-Duran E.. (2023). Ceragenins exhibit antiviral activity against SARS-CoV-2 by increasing the expression and release of type I interferons upon activation of the host’s immune response. Antivir. Res. 217:105676. doi: 10.1016/j.antiviral.2023.105676 37481038

[ref72] Tanaka D. Miyasaki K. Lehrer R. (2000). Sensitivity of Actinobacillus actinomycetemcomitans and Capnocytophaga spp. to the bactericidal action of LL-37: a cathelicidin found in human leukocytes and epithelium. Oral Microbiol. Immunol. 15, 226–231. doi: 10.1034/j.1399-302x.2000.150403.x, PMID: 11154407

[ref73] Tang J. X. Wen Q. Bennett A. Kim B. Sheils C. A. Bucki R.. (2005). Anionic poly (amino acid) s dissolve F-actin and DNA bundles, enhance DNase activity, and reduce the viscosity of cystic fibrosis sputum. Am. J. Phys. Lung Cell. Mol. Phys. 289, L599–L605. doi: 10.1152/ajplung.00061.2005 15964901

[ref74] Taylor-Cousar J. L. Robinson P. D. Shteinberg M. Downey D. G. (2023). CFTR modulator therapy: transforming the landscape of clinical care in cystic fibrosis. Lancet 402, 1171–1184. doi: 10.1016/S0140-6736(23)01609-4, PMID: 37699418

[ref75] Tokajuk J. Deptuła P. Chmielewska S. J. Skłodowski K. Mierzejewska Ż. A. Grądzka-Dahlke M.. (2022). Ceragenin CSA-44 as a means to control the formation of the biofilm on the surface of tooth and composite fillings. Pathogens 11:491. doi: 10.3390/pathogens11050491, PMID: 35631012 PMC9143991

[ref76] Tomás M. Doumith M. Warner M. Turton J. F. Beceiro A. Bou G. (2010). Efflux pumps, Opr D porin, amp C β-lactamase, and multiresistance in *Pseudomonas aeruginosa* isolates from cystic fibrosis patients. Antimicrob. Agents Chemother. 54, 2219–2224. doi: 10.1128/AAC.00816-09, PMID: 20194693 PMC2863613

[ref77] Tomkiewicz RP Kishioka C Freeman J Rubin BK. DNA and actin filament ultrastructure in cystic fibrosis sputum. Cilia, mucus, and mucociliary interactions New York: Dekker. (1998): 333–341.

[ref78] Turcios N. L. (2020). Cystic fibrosis lung disease: an overview. Respir. Care 65, 233–251. doi: 10.4187/respcare.06697 31772069

[ref79] Turner K. H. Wessel A. K. Palmer G. C. Murray J. L. Whiteley M. (2015). Essential genome of *Pseudomonas aeruginosa* in cystic fibrosis sputum. Proc. Natl. Acad. Sci. 112, 4110–4115. doi: 10.1073/pnas.1419677112, PMID: 25775563 PMC4386324

[ref80] White R. Woodward S. Leppert M. O’Connell P. Hoff M. Herbst J.. (1985). A closely linked genetic marker for cystic fibrosis. Nature 318, 382–384. doi: 10.1038/318382a0 3906407

[ref81] Wu M. Maier E. Benz R. Hancock R. E. (1999). Mechanism of interaction of different classes of cationic antimicrobial peptides with planar bilayers and with the cytoplasmic membrane of *Escherichia coli*. Biochemistry 38, 7235–7242. doi: 10.1021/bi9826299, PMID: 10353835

[ref82] Yagel Y. Sestito S. Motro Y. Shnaiderman-Torban A. Khalfin B. Sagi O.. (2020). Genomic characterization of antimicrobial resistance, virulence, and phylogeny of the genus Ochrobactrum. Antibiotics 9:177. doi: 10.3390/antibiotics9040177, PMID: 32294990 PMC7235858

[ref83] Zabner J. Smith J. J. Karp P. H. Widdicombe J. H. Welsh M. J. (1998). Loss of CFTR chloride channels alters salt absorption by cystic fibrosis airway epithelia in vitro. Mol. Cell 2, 397–403. doi: 10.1016/S1097-2765(00)80284-1, PMID: 9774978

[ref84] Zampoli M. Morrow B. Paul G. (2023). Real-world disparities and ethical considerations with access to CFTR modulator drugs: mind the gap! Front. Pharmacol. 14:1163391. doi: 10.3389/fphar.2023.1163391, PMID: 37050905 PMC10083423

